# County Hospital

**Published:** 1873-09

**Authors:** 


***County Hospital.***

After much persuasion, and, rather, too, mature deliberation, the Board of Supervisors of Cook County, have at last determined upon and selected a site as a preliminary step to the erection thereupon of a new County Hospital.

The site selected, the corner of 12th street and Ashland avenue, in what is now the southwest corner of the city, is, we believe, one whose excellence will become more and more apparent with the lapse of time.

Aside from its general advantages in relation to the whole city, it will provide hospital accommodation for that district, the West Side, where it is most especially needed and entirely wanting hitherto. The South and North Sides of the city are at present comparatively well supplied with hospitals, while the West, although containing the largest area, the largest population, and the greatest number of the classes of population more especially requiring hospital accommodation, has none whatever.

While there has been good ground of complaint against the Board of County Commissioners for the delay in making their selection of this site, we are disposed to overlook their past delinquencies in this regard, on the condition that they will display equally good judgment in the adoption of a plan for the building. Let us have no more mediæval castles, with

> "Massive towers and donjon keep,"

with

> "Flanking walls that round them sweep,"

and

> "Dungeons dark where" *patients* "weep."

These are things of the past, which sanitary science repudiates. Too much money has already been squandered in this city upon these illustrations of mediæval art, and sanitary nescience, and let us fervently pray that no more be contributed to the construction of these death-traps.

We commend to the careful consideration of the supervisors the suggestions of Dr. John M. Woodworth, Supervising Surgeon of the Marine Hospital, concerning the erection of Pavilion Hospitals, roughly built, of cheap material, with due attention to ventilation and cleanliness made necessary by the demands of sanitary science, which could be destroyed as soon as they should become foul and unhealthful, and rebuilt many times at a less cost than is now required to construct an ordinary hospital.

Let every physician in the community use his influence to prevent a great waste of public money and a great injury to public health which would inevitably accrue from the rejection of modern ideas in the construction of the new County Hospital. Let it be impressed upon the minds of the commissioners that the object of the hospital is not to enrich contractors, not to be a monument of the ignorance and stupidity of its constructors, but to preserve public health. H.

